# Viewpoints from families for improving transition from NICU-to-home for infants with medical complexity at a safety net hospital: a qualitative study

**DOI:** 10.1186/s12887-019-1604-6

**Published:** 2019-07-05

**Authors:** Ashwini Lakshmanan, Katrina Kubicek, Roberta Williams, Marisela Robles, Douglas L. Vanderbilt, Christine B. Mirzaian, Philippe S. Friedlich, Michele Kipke

**Affiliations:** 1 Fetal and Neonatal Medicine Institute, Division of Neonatal Medicine, Children’s Hospital Los Angeles, Keck School of Medicine, University of Southern California, 4650 Sunset Boulevard, MS #31, Los Angeles, CA 90027 USA; 2 Department of Preventive Medicine, Keck School of Medicine, University of Southern California, Los Angeles, CA USA; 3 Leonard D. Schaeffer Center for Health Policy and Economics, University of Southern California, Los Angeles, CA USA; 4 USC Gehr Family Center for Health Systems Science, Keck School of Medicine, University of Southern California, Los Angeles, CA USA; 5 Division of Research on Children, Youth and Families, Children’s Hospital Los Angeles, Los Angeles, USA; 6 Division of Cardiology, Children’s Hospital Los Angeles; Keck School of Medicine, University of Southern California, Los Angeles, CA USA; 7 Division of General Pediatrics, Children’s Hospital Los Angeles, Keck School of Medicine, University of Southern California, Los Angeles, CA USA; 8 Saban Research Institute, Children’s Hospital Los Angeles, Keck School of Medicine, University of Southern California, Los Angeles, CA USA

**Keywords:** Transition, Early intervention, SSI, Prematurity, High-risk infant, Post-discharge, Outcomes

## Abstract

**Background:**

We have limited information on families’ experiences during transition and after discharge from the neonatal intensive care unit.

**Methods:**

Open-ended semi-structured interviews were conducted with English or Spanish- speaking families enrolled in Medicaid in an urban high-risk infant follow up clinic at a safety-net center, which serves preterm and high-risk term infants. We generated salient themes using inductive-deductive thematic analysis.

**Results:**

Twenty-one participants completed the study. The infant’s median (IQR) birth weight was 1750 (1305, 2641) grams; 71% were Hispanic and 10% were Black non-Hispanic; 62% reported living in a neighborhood with 3-4th quartile economic hardship. All were classified as having chronic disease per the Pediatric Medical Complexity Algorithm and 67% had medical complexity. A conceptual model was constructed and the analysis revealed major themes describing families’ challenges and ideas to support transition centered on the parent-child role and parent self-efficacy. The challenges were: (1) comparison to normal babies, (2) caregiver mental health, (3) need for information. Ideas to support transition included, (1) support systems, (2) interventions using mobile health technology (3) improved communication to the primary care provider and (4) information regarding financial assistance programs. Specific subthemes differed in frequency counts between infants with and without medical complexity.

**Conclusions:**

Families often compare their preterm or high-risk infant to their peers and mothers feel great anxiety and stress. However, families often found hope and resilience in peer support and cited that in addition to information needs, interventions using mobile health technology and transition and financial systems could better support families after discharge.

**Electronic supplementary material:**

The online version of this article (10.1186/s12887-019-1604-6) contains supplementary material, which is available to authorized users.

## Introduction

Approximately 10 % of infants in the United States are hospitalized in the neonatal intensive care unit (NICU) [1, 2]. These infants often join the population of children with “medical complexity” [3, 4]. Medical complexity has been classified using the Pediatric Medical Complexity Algorithm (PMCA) into 3 groups, “complex chronic disease, noncomplex chronic disease and not chronic disease” [5]. Families caring for high-risk infants face multiple challenges in meeting their child’s health needs, which are pronounced in underserved populations. For example, Hispanic and Black families are less likely to be referred to high-risk infant follow up programs [6] and Black families are five to eight times less likely to follow up at the 24 month visit in early intervention programs [7]. Disparities in transition-to-home and follow-up for families who have limited English proficiency and/or are minorities or low-income exist and may be reduced by engaging families in the process of re-designing transition of care from NICU-to-home [3, 4, 8–10].

A recent publication in *Pediatrics*, “Building systems that work for children with complex health care needs,” [11] outlines the importance of involving families “on the front lines” in the designing of the care-coordination of a high-risk infant [12]. The use of family expertise is essential to improving health care delivery [13]. However, only a few studies have engaged low-income, minority and/or non-English speaking families in this process to understand their perspective [14–16]. Some issues that families have previously identified were a lack of awareness of community resources, fragmented service lines, and provider organizations varying in their ability to meet linguistic and cultural needs of different individuals and families [17–22].

While there have been studies published on caregiver perceptions of transitioning to home in older children from the medical or surgical unit of a hospital [23, 24] and from the NICU with a more homogenous sample, [25] there has been little published on transitioning infants with medical complexity home from the NICU at a safety net hospital. Our research question was, “What ideas do families with infants with medical complexity (many of whom are low-income and/or minorities) have to support parents in the transition to home from the neonatal intensive care unit (NICU) at a safety net hospital?” To better understand the perspectives of families regarding follow up after NICU discharge, in this qualitative study, [1] we explored domains such as caring for the infant at home including how health care is received and perceived and challenges and ideas to support transition from NICU-to-home and [2] examined if they differed by medical complexity.

## Methods

### Research team and reflexivity

The author (A.L.), a neonatologist, and research staff with qualitative methods experience conducted the interviews. The authors had familiarity with the patients history and diagnoses prior conducting the interviews. The participants were aware of the authors’ goals and interviewer characteristics.

### Study design and theoretical framework

This was a qualitative prospective study that utilized open-ended semi-structured interviews. The methodological orientation used for this study was inductive-deductive thematic analysis [26]. The conceptual model was derived from the Kenner Transition Model as subsequently described [27].

### Participant selection and setting

The participants were recruited using purposive, typical case sampling. The participants were approached using face-to-face recruitment. Participants were recruited from a high-risk infant follow up clinic at a quaternary urban safety-net children’s hospital in Los Angeles that serves primarily a Medicaid population in the spring and summer of 2017. This clinic provided multidisciplinary follow up for infants with gestational age < 32 completed weeks or birth weight < 1500 g or infants who received extracorporeal membrane oxygenation or therapeutic hypothermia or had other problems that could result in neurologic abnormality.

We included English or Spanish speaking parents if enrolled in Medicaid with infants with complex health care needs. The interview was conducted by telephone and the participant was given an incentive on completion of the interview. The Children’s Hospital Los Angeles (CHLA) human subjects protection program approved the study protocol. Of the 30 eligible participants, 21 participated. We finished recruitment once thematic saturation was reached [28].

### Data collection

We approached study participants in clinic and then scheduled an open-ended semi-structured telephone interview. Either a researcher or research assistant with experience in qualitative interviewing performed the interviews. A literature review and key stakeholder interviews with neonatologists, developmental pediatricians, clinical care coordinators and nurses informed the interview guide (Additional file 1). During data collection, we refined the guide in an iterative fashion. The interview included the following domains: (1) Caring for the infant at home including how health care is received and perceived, (2) How community based resources are used, (3) Health care costs, and (4) challenges and ideas to support transition from NICU-to-home. Interviews were recorded on a digital recorder and then transcribed by a service. When participants finished their interview, they received a $40 gift card. Demographic data and data about the infant’s health were extracted from the medical record.

### Data analysis

Demographic and clinical variables were analyzed via descriptive statistics in SAS, v. 9.4 (SAS Institute, Cary, NC, USA).

Transcripts were imported into ATLAS.ti (v 1.5.4, Scientific Software, Berlin, Germany). We used an inductive-deductive thematic analysis approach; an iterative process of coding to identify the patterns between concepts [29, 30]. A team of three people, with experience in neonatology (A.L.) and qualitative research methods in pediatrics (M.R. and K.K.) reviewed the transcripts. Two coders (A.L. and M.R.) shared initial coding duties and a third coder, (KK) double – coded interviews to increase validity. The codebook was modified as necessary and discrepancies were resolved through consensus and we kept an audit trail. Like Enlow, et al., we developed a set of codes from repeating themes in the data and conducted co-coding until we had consensus [28]. We also used axial coding using inductive and deductive techniques. The ultimate codebook contained 26 codes and 24 sub-codes. We produced 189 pages of transcripts, which were read by the members of the research team (A.L., M.R., K.K.). In total, 423 unique excerpts were coded between 1 and 16 codes each. Subsequently, we placed themes and subthemes in context of infants with chronic complex vs. chronic non-complex conditions. We looked to see if specific issues were raised by a particular group.

## Results

### Demographics

Twenty-one of the 30 eligible participants (20 mothers, 1 father) provided consent and completed the study. As outlined in Table 1, of the participants who completed the interviews, the infant’s median (IQR) gestational age was 31.8 (28.7, 34.5) weeks’ gestation and corrected gestational age at presentation was 6 (6, 12) months. The median (IQR) length of stay for the index hospitalization (NICU hospitalization) was 83 (31, 135) days. Notably, 47% of infants required medical equipment. All infants had chronic disease as classified by the Pediatric Medical Complexity Algorithm (PMCA) and 67% were noted to have medical complexity [5]. Details of diagnoses and characterization are included as Additional file 2: Table S1. Median (IQR) maternal age was 26 (23, 27) years. It was the first child for 43% of mothers, 71% were Hispanic, 10% were Black non-Hispanic, 14% White non-Hispanic, 5% Asian, and 62% reported living in a neighborhood with 3-4th quartile economic hardship. Moreover, 57% of participants lived ≥20 miles from the infant follow up program clinic; in fact, three resided more than 60 miles from the clinic. Of note 100% of participants reported owning/using mobile technology (smartphone, tablet with internet access).

**Table 1:** Infant and Parent Demographics (*n* = 21)

	n (%) or Median (IQR)
Infant demographics	
Race/Ethnicity, n (%)	
Hispanic	15 (71)
Black non-Hispanic	2 (10)
White non-Hispanic	3 (14)
Asian	1 (5)
Gestational age (weeks), median (IQR) if preterm	31.8 (28.7, 34.5)
< 32 weeks, n (%)	9 (43)
< 28 weeks, n (%)	2 (9.5)
Term infants being followed in high risk infant follow up clinic, n (%)	4 (19)
Birth weight (grams), median (IQR)	1750 (1305, 2641)
Chronologic Age (months), median (IQR)	8 (7, 13.7)
Corrected gestational age (months), median (IQR)	6 (6, 12)
Length of stay of hospitalization in NICU (index hospitalization), days, median (IQR)^a^	83 (31, 135)
Time from discharge (months), median (IQR)	6.5 (4.25, 9.5)
Infants with medical complexity^b^ (Diagnoses list available in Appendix)	
Complex Chronic Disease (C-CD), n (%)	14 (67)
Non Complex Chronic Disease (NC-CD), n (%)	7 (33)
Number of medications used after discharge, median (IQR)	4 (1, 5)
Number of medical subspecialists after discharge, median (IQR)	2 (1.7, 5)
Using durable medical equipment^c^, n (%)	10 (47)
Oxygen	5 (24)
Feeding tube (gastrostomy/jejunostomy)	7 (33)
Tracheostomy/Ventilator dependence	1 (5)
Using occupational therapy, n (%)	9 (43)
Using physical therapy, n (%)	7 (33)
Bayley Scores (if > 6 months), median (IQR)	
Composite Cognitive	95 (29, 101)
Composite Language	82 (70, 91)
Receptive Language (scaled score)	8 (4, 9.2)
Expressive Language (scaled score)	7 (4.7, 7.2)
Composite Motor	75 (70, 92)
Fine Motor (scaled score)	8 (3.5, 8.5)
Gross Motor (scaled score)	4.5 (2.7, 7.2)
Maternal and family demographics	
Age (years) at delivery, median (IQR)	26 (23, 27)
Parity status, n (%)	
First child	9 (43)
Siblings	6 (29)
Missing values	6 (29)
Primary Language, n (%)	
English	17 (81)
Spanish	4 (19)
Neighborhood equity^d^, n (%)	
Home zip code in service planning area (SPA) with 3th quartile economic hardship index	4 (19)
Home zip code in service planning area (SPA) with 4th quartile economic hardship index	9 (43)
Distance from home zip code to High Risk Infant Follow Up Clinic (miles), median (IQR)	20.2 (11.2, 27.8)
<10 miles, n (%)	5 (24)
≥10 miles to < 20 miles, n (%)	4 (19)
≥20 miles, n (%)	12 (57)
Medicaid/California Children’s Services, n (%)	21 (100)
Receiving income assistance if eligible, n (%)	7 (33)
Owning a smartphone or tablet with internet capability, n (%)	21 (100)

^a^Length of stay represents number of days (if transferred to higher level of care)

^b^Defined by Pediatric Medical Complexity Algorithm (version 3.0) (measurement tool accessed at: https://www.seattlechildrens.org/research/centers-programs/child-health-behavior-and-development/labs/mangione-smith-lab/measurement-tools/)

^c^Medical equipment: gastrostomy tube, tracheostomy tube, helmet, oxygen, colostomy

^d^Service planning area 6, 7, 8 (4th and highest quartile of economic hardship index)

### Conceptual model

**Fig. 1:**
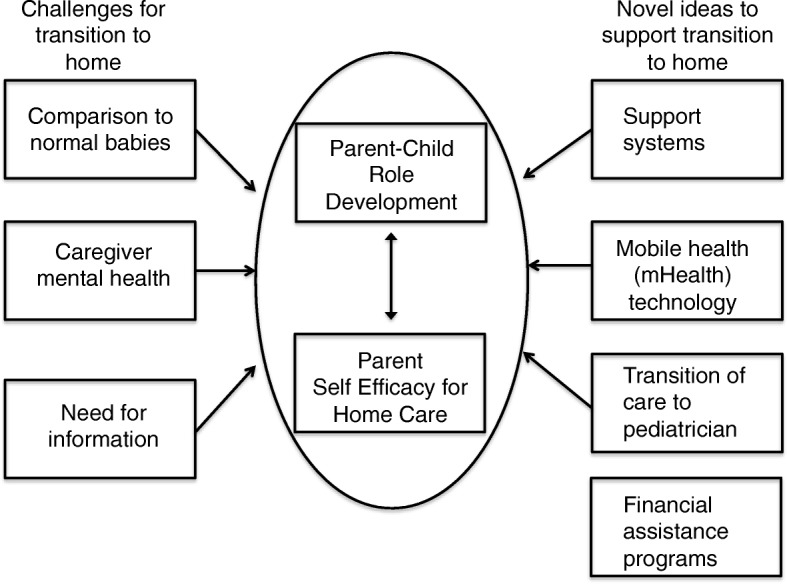
Conceptual Framework for outlining families’ priorities for transitioning low-income families with infants with medical complexity home from the NICU

A conceptual model was constructed and derived from the Kenner Transition theoretical framework as illustrated in Fig. 1 [27]. This model classifies parental concerns and challenges “into 5 categories: Informational Needs, Stress and Coping, Grief, Social Interaction and the Parent-Child Role Development.” The features in this model are “interrelated and reciprocal” [27]. Boykova, et al. described that each of the categories in the model influence each other. For example, informational needs is at the center of the model and informs and affects the other categories. This model emphasizes that transition is “a process” of change and not a “product of change.” Therefore by viewing each of these categories as connected and not disparate, we can appropriately support proposed interventions for this group of patients. Specifically in our conceptual model, parent-child role development and self-efficacy both inform and affect each other and may be influenced by challenges and ideas to improve transition.

### Themes

The analysis revealed seven major themes in two categories describing families’ challenges and ideas to support transition centered on the parent-child role and parent self-efficacy. It is important to note that we generated the theme of parent self-efficacy rather than parent confidence. Vance et al. distinguished between the two concepts as the following: parenting self-efficacy may be conceptualized as the “global” parent belief in their ability to “carry out parenting tasks” while parenting confidence is defined as the judgment a parent holds about “their ability” to be successful with domain-specific parenting tasks. However, after extensive literature review, Vance did note that ultimately these terms may interchangeable [31].

The challenges were: (1) comparison to normal babies, (2) caregiver mental health, and (3) need for information. Ideas to support transition included, (1) support systems, (2) mobile health technology (3) improved transition to the primary care provider and (4) financial assistance programs. Specific subthemes differed in frequency counts between infants with and without medical complexity. Representative quotations are presented in Table 2 and differences in subthemes are presented in Table 3. Major differences included that families with medical complexity had more information needs (for durable medical equipment, orientation to vendors and early intervention services), turned to family for support and requested mobile health technology communication tools. Families with children without medical complexity reported wanting improved transitions/handoffs to their primary care providers (pediatricians).

**Table 2:** Themes and Illustrative Quotations

Themes	Sub Themes	Quotations
Parent-child role relationship		“I mean I have 3 other kids but it feels like being a first time parent in a lot of ways. It’s just, his health needs are so different that I often feel like I don’t know what the right answer is and how to do things correctly with him…Even teaching him to eat is very, very different than my other kids. I feel like everything is just kind of a different path.”
Parent self-efficacy		“I think it should be started, they should start talking to parents about this you know the, first week or so that the baby is there, to start introducing to the parents how life is gonna change. If they give it to the parent when the baby’s discharged and I get thrown all this information, parents will not even listen. You got a brand new baby and you gotta figure all this out. It’s kinda like uh overwhelming. It’s like introduce the parent little by little while the baby’s in the hospital, these are gonna be the things the baby’s gonna need, be aware of things that you know might help, um start contacting different departments…for the parent to be aware and be involved you know and start researching before the baby actually comes home. And you know then it probably relieves the parent of a lot of stress and a lot of um anxiety, you know, knowing that you have this extra help, that you’re not alone, and you’re not trying to figure this baby out by yourself.”
Challenges to transition		
Comparison to normal babies	Feeling babies are more fragile Prone to illness More critically ill Feeling like their development is behind	“Because of everything I saw him go through inside the NICU. When we first got him, he didn’t look like a normal baby. He looked like deformed. And it’s just different things and things that other babies go through, all that other stuff as well…it was a tough experience.” “I mean I have 3 other kids but it feels like being a first time parent in a lot of ways. It’s just, his health needs are so different that I often feel like I don’t know what the right answer is and how to do things correctly with him…Even teaching him to eat is very, very different than my other kids. I feel like everything is just kind of a different path.” “Because other, naturally other children are healthy so they don’t need the extra care, they don’t need to go to the hospital so much often. And with my baby, it’s, I have to make sure that he’s clean and whoever touches him has to wash their hands. I have to make sure that the colostomy bag is clean. And I take him to therapy a lot, every week. And so it’s, it’s you know so different between a healthy baby and an unhe-, a baby who’s been in the NICU and has had problems.” “I guess there’s one of my main concerns is that I know my baby’s a little bit delayed. So I’m just afraid he might not catch up even though I’m doing everything I can so he can catch up with OT, PT, and infant stimulation. I just feel like maybe my baby’s not fast enough.” “And I get scared by the time he reaches the age to go to school, I don’t want him to get bullied or kids laugh at him because he’s probably still a little bit delayed.”
Concern for mental health	Feeling more sad Anxious	“I mean every now and then there’s kinda like the feeling of anxiety and, and a bit of depression because you know I’m home all day, not really any interaction and it’s kinda stressful taking care of an ill child who, you know, you’re trying to do your best for but you feel like your best kinda isn’t enough.” “Well, one thing about being in the NICU, especially if you’re in there for a long time um, stress and anxiety and depression really, really can take ahold of you. I would say definitely should be a part of it is to teach parents how to occupy their time in a different way, even if they’re at the hospital, do some scrap booking or take pictures or decorate your baby’s room, do something that’s gonna you know lighten up the moment” “Um you know [I needed] also some advice and help to not feel so sad.” “Before I had my baby, I was planning on going back to work. But since my [child] needs special care and there’s no one here at home [who] can help me with that, I’ve been doing everything on my own. And at the beginning, it was a little bit hard because like I was a single mom and new baby, and you know, I, I, I was doing everything on my own. I was pretty exhausted all the time and I started to become a little bit depressed.”
Information Needs	Medical equipment Orientation to services ahead of time Directory of services and vendors	“getting to know about the services that are available for you and for us involved with care that he needs, actually being that he’s considered a special needs kid, which I didn’t find out until about a month and a half ago when the insurance was having all these issues...he was considered special needs kid. So, like providing parents with the correct information, not just being here’s this video of how to care for him at home.” “Um, you know, just resources for parents that are, you know, kind of, might be new to it. ‘Cause for us it was a shock. And I think I would have loved for someone to explain to us the process a little bit more thoroughly. Kind of sit there and, and, kind of guide us through the process as far as you know, what can happen. You know, what, you know, what are the possibilities. …just being able to understand, um, all the possibilities of the baby being in the NICU, whether they’re good or bad. And then just, just guiding us through any programs that could be available.” “Honestly when we first took him home, it was all very, very overwhelming trying to figure out how to honestly use the equipment and he was just much sicker at the beginning too. So try to manage his health and then trying to understand all these different programs that are out there, which are very beneficial but they’re not easy to navigate. Um, yeah honestly even if [the hospital] had recorded videos kind of explaining a little bit more about how some of these things work, like explaining what SSI is and what IHSS is, all these programs I’d heard about but didn’t really know about. … I mean we do have a social worker now who’s helpful with that kind of stuff, but yeah at the beginning we weren’t really plugged in with a social worker who could answer those sorts of questions, so something like that could be helpful just to explain to parents how to do all this stuff.” “Honestly my husband still isn’t that comfortable with the feeding tube. I do most of that, he’s still not that comfortable with the g-tube and with the pump. Honestly, if you had basic videos on that stuff online that would help. To kind of be like refreshers and for grandparents and people like that. That could be very helpful, like basic videos, how to, like how do you use a suction machine. I mean you need to learn in person but reminder stuff that would be great” “I guess having just initially left the NICU, I was like oh man I don’t know who to contact for questions about her g-tube, questions about this, questions about that. Because they always you know come and see you after these procedures are done and things. And you meet the doctor for a few minutes, you ask and you have some questions, they come and check on you maybe once before you’re discharged and then after that you don’t really see them until the next appointment. But it’s like well what if something comes up between your discharge and the appointment? You don’t know who to call except to say oh my daughter has a g-tube and there might be an issue with it. So having you know phone numbers, basically a directory to different departments, different doctors, what their hours might be or when offices are closed or what not or you know and email addresses, things like that would be just initially so helpful. Because otherwise I’ve kind of had to wait it out. Sometimes I’m like well luckily she’ll see that doctor in 3 or 4 days so I guess I’ll just wait until then you know.” “I think it’d probably be helpful to have like a packet when you leave with just a bit of summary of all these different programs, like explanations about what they are. I was confused about EI and regional center and all that stuff for quite a while. Like not just contact number but actually like a brief description of what they are for.”
Ideas to support transition		
Looking for support systems	Peer support Family support Really looking for families going through the same thing	“Counseling, or any, comforting or something, you know… that’s one thing I saw… frequently in the NICU. Is that, you know, no one really talked about, you know, this is a possibility. Or, you know, this is what can happen. You know, there’s a chapel downstairs if you’d like to talk to someone. Or, you know, we have a patient specialist that can come up and if you have any questions, or stuff like that” “because you’ve been through so much and sometimes um, um like you need [to] like talk to somebody about your feelings” “[Having] a support group where you could talk to other parents would be useful. Okay, so kind of having like other people’s experience, and like knowing that you’re not going through it alone” “I am on some groups on, like, online, with other parents, and we all ask questions about, oh my kid has been having these symptoms and then it’s the same diagnosis that the other babies have, and they might be similar and you can sort of get an idea what’s going on.” “What might have helped you learn more about taking care of your baby?” “more like actual parent experiences of their babies being in the NICU how their journey to go like being there every day or trying to supply your baby with milk, breast milk and just, just like the whole experience I think would be really good.”
Including mobile technology and internet into discharge materials	Video ChatHealth PortalVideos/materials available on lineWould also like an in person supplementHotline	“I love it. I usually will Google specific sites, usually there is specific web pages where I can go and find all the information that, uh, is good for me and for the baby, I will always be grateful.” “Yeah, being able to receive stuff like via text even and email it’s awesome.” “And you know if I receive a text or an email I could check it and I’ll be like okay um you know I have an appointment or something like that. It would be, it would be really, really,an app for me it would be um, good.” “I think sometimes video chat would be an awesome way if they had like with her g-tube feedings, I could kind of be like, hey am I doing this right? You know, or can you see what I see? Like I am concerned about this you know, so I should bring her in? Like you know that kind of thing that would be awesome. Just the amount of technology that we have access to is phenomenal.. Sometimes you know doctors will ask me, ‘oh well what does her stool look like’? Well, how do I explain it? It looks like cement that’s not dried yet and it’s green and it’s this color, it’s this texture. So it’s like uh can I just show you? And that’s what I do, sometimes I have to take a picture and I send it through email.” “For me, the best way would be either video chat or like on the phone or something, because you know I can’t always make it down to meet up with someone in person. … and I’m saying speaking to someone is better so you have a full understanding of what’s going on versus like email or text or something like that” "Sometimes [it’s]hard to keep up with all the doctors and all the, appointments, so being able, … to see it, and you know, probably be able to read up on the notes, because sometimes we forget to get the discharge papers, or you know, follow up notes, and we kind of forget them, so probably better if they would just do it all online, so we can just look at it, and you know, print it out if we need it,
Improving transition of care to pediatrician		“And our pediatrician used to be um one of the doctors that used to see her at the NICU. So that was an advantage and she pretty knew what she needed, um she didn’t need to be asking questions, she pretty much knew exactly what she needed or what’s good for her.”
Identifying financial assistance programs		“I signed up last year but it was the longest process of my life. My worker kinda dropped the ball for a minute and didn’t really do what, what’s supposed to be done. And didn’t you know send out the paperwork that I needed to fill out and, and send back to her or whatever. So from May, I signed up in May I didn’t get anything until December.”

**Table 3:** Counts of study participants identifying specific challenges and ideas to support transition to home from the neonatal intensive care unit of infants with medical complexity

Challenges to transition	Total (*n* = 21)	Complex chronic disease (*n* = 14)	Non-complex chronic disease (*n* = 7)
Comparison to normal babies	19	12	7
Feeling babies are more fragile	7	3	4
Prone to illness	12	9	3
Feeling like their development is behind	7	4	3
Concern for caregiver mental health	9	6	3
Anxiety	6	4	2
Depression	9	6	3
Information Needs	12	10	2
Durable Medical Equipment/medications	11	9	2
Orientation to services/vendors	7	5	2
Orientation to specialists	7	5	2
Accessing Early Intervention Services	12	10	2
Ideas to support transition			
Support systems	9	8	1
Peer support	4	3	1
Family support	8	8	0
Including mobile health technology	10	7	3
Online Chat (telehealth)	4	4	0
Online forums	2	2	0
Videos/materials available on line	4	2	2
Communication via smartphone and texting	10	7	3
Improved transition of care to primary care provider (pediatrician)	11	6	5
More support for identifying financial assistance programs	11	8	3

### Emphasis on parent-child role and parenting self-efficacy

Parents cited that they sometimes had concerns with their parent-child role development and confidence, “I mean I have three other kids but it feels like being a first time parent in a lot of ways. It’s just, his health needs are so different that I often feel like I don’t know what the right answer is and how to do things correctly with him…Even teaching him to eat is very, very different than my other kids. I feel like everything is just kind of a different path.”

Others were concerned about their level of self-efficacy to transition from NICU-to-home to care for their children at home. For example, one parent stated, “I think it should be started, they should start talking to parents about this you know the first week or so that the baby is there, to start introducing to the parents how life is gonna change. If they give it to the parent when the baby’s discharged and I get thrown all this information, parents will not even listen. You got a brand new baby and you gotta figure all this out. It’s kinda like uh overwhelming. It’s like introduce the parent little by little while the baby’s in the hospital, these are gonna be the things the baby’s gonna need, be aware of things that you know might help, um start contacting different departments…for the parent to be aware and be involved you know and start researching before the baby actually comes home. And you know then it probably relieves the parent of a lot of stress and a lot of um anxiety, you know, knowing that you have this extra help, that you’re not alone, and you’re not trying to figure this baby out by yourself.”

### Challenges to transition

#### Theme 1: comparison to normal babies

Parents often compared their babies to their term peers. Sub-themes included parents’ feeling their babies were more fragile, prone to illness, critically ill and belief that the baby’s development was behind. For example, parents shared: “I guess one of my main concerns is that I know my baby’s a little bit delayed…I feel like my baby is not fast enough” and “I’m afraid he might…get bullied or kids laugh at him because he is a little bit delayed.” Parents also felt that being a parent to a baby who was hospitalized in the NICU was very different than their other experiences, “I mean I have three other kids but it feels like being a first-time parent in a lot of ways.” Families with infants with complexity cited the subtheme “prone to illness” while more families with infants without complexity cited the subtheme, “feeling babies are more fragile.”

#### Theme 2: concern for maternal mental health

Mothers expressed concern for their own mental health and reported frequently feeling sad and anxious: “there’s kinda like the feeling of anxiety and depression because you know I’m home all day not really any interaction and it’s kinda’ stressful taking care of an ill child, who you’re trying to do your best but you feel like your best kinda’ isn’t enough.”

#### Theme 3: information needs

Families highlighted the need for more knowledge about taking care of a child with possible special health care needs with information that is consistent and clear. They also wanted to have better communication with their medical provider. They suggested better orientation to medical equipment, services ahead of time and perhaps even creating a directory of services and vendors. For example, families suggested it was important “to get to know the services that are available for you…being that he’s considered a special needs kid.” In addition to medical services, families wanted to learn more about ancillary and community-based services as well, “Honestly, if we had videos explaining a little bit more about how some of these things work, like explaining what SSI or IHSS is…explaining to parents how to do all this stuff.” Families with children with medical complexity more frequently requested more information than families with children without medical complexity, especially about early intervention and durable medical equipment (10 of 14 families with children with medical complexity versus 2 of 7 families with children without medical complexity).

### Novel ideas to support transition

#### Theme 1: looking for support systems

Families pointed out that support systems may be integral to coping and improving life after NICU discharge. Sub-themes included peer and family support and, particularly, looking for families going through the same thing, “you’ve been through so much and sometimes like you need to talk to somebody about your feelings” or “talking to other moms that have a premature baby.” Interestingly, families with children with medical complexity seemingly turned more to family support than those without medical complexity (8 of 14 families with children with medical complexity vs. none of the families with children without medical complexity).

#### Theme 2: incorporating mobile technology into discharge planning

Families emphasized the use of mobile health technology into discharge planning; ideas included video chats, health portals, online videos/material resources and a hotline. For example, parents shared, “I think video chat would be awesome…can you see what I see for example with g-tubes?” Similarly, families suggested sometimes “we forget our discharge papers and it would be better if they would do it all online so we can just look at it.”

#### Theme 3: importance of high-quality transitions to primary care

More families without medical complexity cited the theme that they would appreciate high-quality transition of care to their pediatrician. For example, a family stated, “And our pediatrician used to be um one of the doctors that used to see her at the NICU. So that was an advantage and she pretty knew what she needed, um she didn’t need to be asking questions, she pretty much knew exactly what she needed or what’s good for her.” Another parent stated, “Well thankfully her pediatrician-she’s helped me [so] much with my baby’s needs so I feel like I’m with enough support from her-she’s really, really good when it comes to my baby’s care.”

#### Theme 4: increased enrollment in financial assistance programs

Families with and without medical complexity cited the importance of enrollment in financial assistance programs and how challenging it could be to sign up for such programs. “I signed up last year but it was the longest process of my life. My worker kinda dropped the ball for a minute and didn’t really do what, what’s supposed to be done. And didn’t you know send out the paperwork that I needed to fill out and, and send back to her or whatever. So from May, I signed up in May I didn’t get anything until December.”

## Discussion

We conducted in-depth interviews with families enrolled in Medicaid after NICU discharge to better understand what challenges and ideas families had to facilitate complex transition for this vulnerable population. With our findings, we highlight the importance of families’ viewpoints on transition and discharge. Families often compare their preterm or high-risk infant to their peers, and mothers feel great anxiety and stress perhaps compounded by postpartum depression. However, families often found hope and resilience in peer support and cited that in addition to health literacy, interventions using mobile health technology and respite/nursing care could better support families after discharge. We also identified that there are different subthemes and constructs that are valued by families with and without children with medical complexity.

The first theme, comparison to “normal babies” identified in our cohort is comparable with several other studies in different populations. Families repeatedly expressed concern about their child as “more fragile” to a healthy term peer and this concept has been generalizable across socioeconomic status [3, 28, 32, 33]. There has been other literature to suggest that this comparison may affect how parents view their feelings of discharge readiness and capability to take care of a child with advanced medical needs. While not observed in this study, there may also be more pronounced feelings of mistrust and disparities in minority families accessing multidisciplinary services such as a medical home, [34, 35] which may further encumber these families, and further investigation is needed. Special attention should be paid to these families to optimize communication with primary care providers and discharge readiness [2, 36, 37].

Mothers who participated in the study also pointed to stress, sadness, anxiety and even depression when taking care of their infant. The literature is ripe with evidence to support that postpartum major depressive disorder (MDD) is pervasive (1 in 5 women) [38] and magnified in mothers of preterm infants, and our findings suggested that women in our sample might be dealing with similar issues [38–41]. Hawes et al. identified that “three potential risk factors for postpartum depressive symptoms include: history of maternal mental health diagnosis, social factors, and perception of readiness at NICU discharge” [38]. The US Preventive Services Task Force and several states have recommended and/or mandated screening for MDD [42]. While detection has improved, maternal outcomes have not because of low rates of treatment engagement [43]. We would suggest integrating mental health referrals early in the index (NICU) hospitalization and perhaps using the venue of a high-risk infant follow up program to assess adherence for mental health follow up.

As is true with many other populations of children with special health care needs, our families were looking for gains in health literacy and knowledge. Unique to our population, is that they are either first-time parents or “first-time parents with children with advanced medical needs,” and they want to learn the “language” of medicine to communicate with their providers. Moreover, fragility in such an insecure medical state may heighten concerns that parents are experiencing. Several studies have examined the disparities in outcomes with parents who are less health literate in pediatric diabetes clinics, emergency departments, or primary care [44–46] and in families of preterm infants [47]. By providing parents with more tools about the medical care of their infant and referrals for social services early in transition to home and thereafter, we may empower families and improve infant outcomes [48].

Families looking for support systems have been a common theme in other populations of children with special health care needs, such as cancer, developmental delay, and autism [49, 50]. Parents in our study were seeking communities that have experienced the same journey as they have. A handful of studies have examined the role of social media websites and forums and their impact on parenting and help-seeking behaviors [49, 51]. For example, Thoren et al. evaluated the content of communication in 1497 Facebook communities dedicated to preterm infants and found that “information sharing” (31%) and “interpersonal support” (53%) were the most common purposes of use of the Facebook groups [52]. Similarly, another study queried families about what content they would share on social media and 79% answered that they would be interested in joining a “native-language online networking site providing: (1) general information on prematurity, (2) explanations of abbreviations commonly used in a hospital setting, and (3) details of common medical problems and the treatment thereof, including the availability of local therapists and follow-up services” [53]. Social media might be particularly useful for fathers as a platform to discuss their concerns and gain emotional support in future studies [54]. It also might be useful for families with CMC to also have similar supports for family members.

Leveraging technology into discharge planning such as mobile health (mHealth) applications, telemedicine, video chat and patient portals may enable follow up in a more flexible manner. This may suit the needs of minority and low-income families who often face additional barriers, such as language, transportation and cultural differences, to attend prescribed follow up. For example in our population, more than half of participants were traveling more than 20 miles for their follow-up appointment. Alderdice et al. recently published a study that reviewed potential websites that families found useful after discharge home [55]. A recent meta-analysis suggested families have generally accepted mHealth interventions while in the NICU [56] which may be translated to the outpatient settings as well. Other studies have supported the use of webcams in the NICU and videoconferencing with families at home [57, 58]. Despite the confines of income, 100% of our participants owned and regularly used a smartphone or tablet with Internet access. Therefore, incorporating discharge materials into a mobile web-based platform may streamline and facilitate follow up. While designing such interventions, we must consider access to service and shared cellphone use and perhaps consider a hybrid program that integrates in-person navigation as well.

Families also cited the importance of a high quality transition or “hand-off” to their pediatrician. Caregivers of infants, especially those without medical complexity in our sample, thought that the communication with the pediatrician in transition is very important and felt more satisfied when the pediatrician was familiar with their child’s case. Some novel ideas that have been published include the feasibility of a cloud-based care plans for providers and families. Desai, et al. found that providers were amenable to participating in these types of coordination [59] and this might be ideal for infants transitioning from several types of NICUs (community based, children’s hospital, etc.) to a primary care provider.

The difficulty of enrolling in financial assistance programs such as supplemental security income (SSI) and transitional aid to families was also salient in our sample. Low-income families who have children with special health care needs still rely on SSI. Several high-risk infants meet criteria by diagnoses (cerebral palsy, stroke or weight/gestational age) and parental income [60]. Several studies have demonstrated that income assistance for families in poverty with children with mental health and disability is crucial for improved outcomes [50, 61–65].

This study also provided some insights on how transition can vary by medical complexity groups. For example, for families with infants without medical complexity, the importance of “hand-off” or communication with the primary care provider was weighted as most valuable. For families with CMC, more information on durable medical equipment, and vendors was useful. Similarly, while both groups appreciated support systems, families with CMC wanted more integration of family. “Tiered quality improvement” efforts for NICU to home transitions based on medical complexity might maximize quality and resource utilization [23].

This study has several limitations. The sampling was purposeful to enroll families receiving Medicaid from a high-risk infant follow up program in an urban quaternary children’s hospital that serves low-income and mostly minority families, so its generalizability may be limited. Therefore, concerns of families with private health insurance are not represented. These families face their own burdens with increased out-of-pocket expenses from medications, medical equipment or even high deductible health plans and separate studies should be conducted to gain their viewpoints [66, 67]. We did, however, reach thematic saturation suggesting that we met the needs of our target population but we mostly recruited low-income Hispanic and Black non-Hispanic families. Values may vary in other racial, ethnic and socioeconomic groups [4]. Finally, since individuals who may have also provided clinical care for the infant in the NICU or clinic conducted some of the interviews, there is a possibility that participants were not as open or honest with their responses.

## Conclusions

Interestingly, similar to other studies [68], we found that there were similarities between what families cited as challenges and what they recommended as suggestions for improvement during the transition process. We found that families often compare their preterm or high-risk infant to their peers, they and mothers feel great anxiety and stress perhaps compounded by.

postpartum depression. However, we identified several ideas that families themselves proposed to improve follow up after NICU discharge including peer support, health literacy, mobile health technology and respite/nursing care. To address the chasm in current healthcare delivery for this population, interventions that might be successful include the development of technologies such as mobile health applications to create “a patient-centered medical neighborhood,” [4] integration of social workers in the care team, in-person visits with community health workers and group visits [4]. Like other transition-to-home programs we believe that these types of interventions may also provide cost-savings [69].

## Additional files

Additional file 1: (Interview Guide). Interview Guide. This is the interview guide that was used during the semi-structured interviews (DOCX 18 kb)

Additional file 2: **Tables S1**. Table of Medical complexity Diagnoses lists). Table that describes medical complexity diagnoses based on the Pediatric Medical Complexity Algorithm (DOCX 18 kb)

## Data Availability

The datasets generated and/or analyzed during the current study are not publicly available due to the data being in transcript form but are available from the corresponding author on reasonable request.

## Abbreviations

NICU: neonatal intensive care unit
SSI: Supplemental Security Income
