# Supplementary material for: Viewpoints from families for improving transition from NICU-to-home for infants with medical complexity at a safety net hospital: a qualitative study
Source: BMC Pediatr. 2019 Jul 5;19:223. doi: 10.1186/s12887-019-1604-6 (PMC6610911; doi:10.1186/s12887-019-1604-6)
Supplement: Supplementary file 1 — (Interview Guide). Interview Guide. This is the interview guide that was used during the semi-structured interviews (DOCX 18 kb) [file 12887_2019_1604_MOESM1_ESM.docx]

**Additional file 1 (Interview Guide)**

**Thank you for participating. The next few questions are about your life at home with your baby.**

1. What has life been like for you at home after your baby was discharged from the neonatal intensive care unit? (Probes: any worry, anxiety, level of support)
   1. Who has been the primary in-home caregiver?
2. What is your home like? (Probes: apartment, house, how many people live at home, type of housing?)

**The next few questions are about your health care and how your receive it.**

1. What are the primary health concerns about your child?
   1. Why do you think taking care of a high risk infant (a baby who has been in the NICU) is different than other children?
2. Do you have a primary care pediatrician to help manage your baby’s medical needs? To what extent PCP manage medical needs? If you don’t, why don’t you have one (insurance)
3. What kind of clinic is it? (Probes: community-based, private office)
4. Tell me about the medical specialists your child has seen (primary health concerns they have about their child) since being home? Examples include neurology, pulmonology, ophthalmology, high risk infant follow up clinic like today?
   1. What made attending these appointments easy? (What facilitated going to these appointments?)
   2. What made attending these appointments difficult? (Probes: transportation, making appointments, paperwork, other process issues)
5. Please tell me what you know about Early Intervention services. (Probes: community based developmental follow up, other names are Regional Center).
   1. Facilitator, impediment for early intervention (developmental delay),
   2. Stigma not coming to terms with that diagnosis
   3. Perception of developmental diagnosis?
   4. Has your baby had his or her first evaluation?
   5. What made attending this follow up easy? (What facilitated going to these appointments?)
   6. What made attending this follow up difficult? (Probes: transportation, making appointments, paperwork, other process issues)
   7. What specialists do you follow with from regional center? Physical therapy, occupational therapy, speech therapy? Any others?

**What were some challenges you faced when you transitioned home from the NICU?**

**The next few questions are about your health care costs and special needs related to your baby.**

1. Has your out of pocket spending for health care for your baby been more than expected? Less than expected?
   1. How much have you spent in the past month on health care related costs?
   2. How much have you spent on transportation alone (gas money, bus fare, etc.
2. Does your baby need to take any medications? Please describe how easy or hard it is to keep up with giving the medications. (Probes: types of medications, schedule, filling prescriptions)
3. Does your baby need any special medical equipment? Please describe how easy or hard it is to keep up with giving the medications. (Probes: types of equipment are used including wheelchair, feeding tube, oxygen, schedule, filling equipment prescriptions)
   1. Probes (How much is covered by insurance)?
4. Do you have a plan in case in there is a medical emergency with the baby?
   1. Are you receiving supplemental security income (SSI)? Do you know what this is and if you are eligible for it?
      1. What made receiving SSI benefits easy? (What facilitated going to the process?)
      2. What made receiving SSI difficult? (Probes: transportation, making appointments, paperwork, other process issues)

**The next few questions are about your home life with your baby related to your other children, sleeping and eating.**

1. If you have other children, how have they coped with having a baby at home? (Probes: Time with other child, understanding that this baby has more special needs)
2. Please tell me about your sleep practices with your baby? (Probes: Do you co-sleep, separate sleep space for the baby, anything in the crib) How did you decide on this practice?
3. Please tell me about your feeding practices with your baby.
   1. If you are breastfeeding, what has made the process easy or difficult?
   2. Are you pumping regularly?
   3. What mix of formula/breastmilk is the baby receiving?

**The last few questions are about ways that we can help after discharge. Do you have ideas how the transition might have been better from NICU to home?**

1. With a lot of families having access to the internet and smartphones, what do you think about receiving medical communication that way? Probes: Text, email, app for coordinator?
2. How do you think having people help you coordinate care after discharge would be useful? (Probes: how would they be useful – in person or phone call or video chat?, printed materials, movies?)
   1. If a program was designed to do this, what materials, information and assistance would be useful?
