# Supplementary material for: Viewpoints from families for improving transition from NICU-to-home for infants with medical complexity at a safety net hospital: a qualitative study
Source: BMC Pediatr. 2019 Jul 5;19:223. doi: 10.1186/s12887-019-1604-6 (PMC6610911; doi:10.1186/s12887-019-1604-6)
Supplement: Supplementary file 2 — Tables S1. Table of Medical complexity Diagnoses lists). Table that describes medical complexity diagnoses based on the Pediatric Medical Complexity Algorithm (DOCX 18 kb) [file 12887_2019_1604_MOESM2_ESM.docx]

**Additional file 2: Table S1 (Table of Medical complexity Diagnoses lists)**

| **Subject Identification and Classification** | **Diagnoses list (P=progressive)** | **Description of qualifying diagnoses (P=progressive)** | **Number of PMCA Diagnoses (Progressive)** | **Use of durable medical equipment** |
| --- | --- | --- | --- | --- |
| 1  C-CD | Q42.3, Q68.8, Q63.2, R62.51, Z93.4, G80.8 (P), F82, P70.1 | Imperforate anus, torticollis, cerebral palsy (P), developmental delay | 4 (1) | Helmet |
| 2  C-CD | Q14.2, Z99.11, G40.919, Z93.1, L92.9, G81.9, G40.109, Q04.5 (P), H52.11, F88, P07.35, H47.9,H50, H47.2, H54.7 | Congenital malformation of optic nerve, epilepsy, hemiplegia, focal epilepsy, megalencephaly (P), disorder of psychological development, cortical visual impairment, esotropia, optic atrophy | 9 (1) | Gastrostomy tube |
| 3 C-CD | P27.1, R09.02, P07.10 | Bronchopulmonary dysplasia | 1 (0) | Oxygen |
| 4 C-CD | P27.1, P07.10 | Bronchopulmonary dysplasia | 1 (0) | Oxygen |
| 5 C-CD | P07.23**,** P27.1,Q68.8, P07.3, H35.113, M43.6 | Bronchopulmonary dysplasia, torticollis | 2 (0) | Oxygen |
| 6 NC-CD | P91.6, P24, R90.89, F82 | Developmental delay | 1 (0) |  |
| 7 C-CD | G93.1 (P), P91.60 | Anoxic brain damage (P) | 1 (1) |  |
| 8 NC-CD | P07.20, H35.109, F88 | Developmental delay | 1 (0) |  |
| 9 NC-CD | R78.81, K55.30, F80.1 | Language developmental delay | 1 (0) |  |
| 10 C-CD | R90.89, P94.2, Z93, F88,R09.02,Q75.3,Q67.3, R06.1, Q68.8 | Developmental delay, plagiocephaly, torticollis | 3 (0) | Tracheostomy |
| 11 NC-CD | E87.2, P91.61, F82 | Developmental delay | 1 (0) |  |
| 12 C-CD | Q39, Q87.89, P07.30, F82, Z93 | Atresia of esophagus, VACTERL, developmental delay | 3 (0) | Gastrostomy tube |
| 13 C-CD | Q37.8, R06.00, P70.1, Q89.7, Z99.11, J96.00, M26.04, G47.33, R09.02, M27.2, T84.7XXA, G47.31 | Cleft palate/lip, multiple congenital anomalies, obstructive sleep apnea, primary central sleep apnea | 4 (0) |  |
| 14 C-CD | I82.402, G00.9, Q79.59 (P), Q04.3(P),Q61.4,F82 | Other congenital malformations of abdominal wall (P), other reduction abnormalities of brain (P), renal dysplasia (P), specific developmental delay | 4 (2) |  |
| 15  NC-CD | P07.1, O60.10X0, F80.1 | Expressive language delay | 1(0) |  |
| 16  C-CD | Z93.1, P27.1,G47.33, Q65.89,Z87.440,P07.3,H35.113,N13.729 | Bronchopulmonary dysplasia, obstructive sleep apnea, vesicoureteral reflux | 3(0) | Gastrostomy tube |
| 17  C-CD | H35.143, Q33.6 (P), R09.02, F88,Z3A.23 | Pulmonary hypoplasia (P), developmental delay | 2(1) | Ventriculoperitoneal shunt, gastrostomy tube, oxygen |
| 18  NC-CD | P24.01, P28.5, F88 | Developmental delay | 1(0) |  |
| 19  NC-CD | H35.109,K21.9,P07.20,  P05.9, F88 | Developmental delay | 1(0) |  |
| 20  C-CD | Q31.5,Z99.81, J84.9(P), R90.89,H90.3,Z89.898,  F80.1,F82 | Interstitial pulmonary disease (P), sensorineural hearing loss, language delay, motor delay |  | Oxygen, gastrostomy tube |
| 21  C-CD | Q39.0, Q42.1, Z93.1, I10, E87.6, Z93.2, J98.51, Q25.1, J18.9, N82.3, H35.113, Q60.0, Q87.89, F88, Q65.89, J86, P07.30 | Atresia of esophagus without fistula, absence of rectum, hypertension, mediastinitis, coarctation of aorta, renal agenesis, VACTERL, developmental delay | 7(0) | Gastrostomy tube |

*(C-CD=Complex Chronic Disease, NC-CD=Non-Complex Chronic Disease)

**Defined using Pediatric Medical Complexity Algorithm (version 3.0, ICD10): https://www.seattlechildrens.org/research/centers-programs/child-health-behavior-and-development/labs/mangione-smith-lab/measurement-tools/)
